# Assessing the impact of antimicrobial stewardship implementation at a district hospital in Ghana using a health partnership model

**DOI:** 10.1093/jacamr/dlad084

**Published:** 2023-07-17

**Authors:** Obed Kwabena Offe Amponsah, Aaron Courtenay, Nana Kwame Ayisi-Boateng, Ahmed Abuelhana, Douglas Aninng Opoku, Lawrence Kobina Blay, Nana Akua Abruquah, Annabella Bensusan Osafo, Charlotte Boachie Danquah, Phyllis Tawiah, Mercy Naa Aduele Opare-Addo, Alex Owusu-Ofori, Kwame Ohene Buabeng

**Affiliations:** Department of Pharmacy Practice, Faculty of Pharmacy and Pharmaceutical Sciences, College of Health Sciences, Kwame Nkrumah University of Science and Technology, Kumasi, Ghana; School of Pharmacy and Pharmaceutical Sciences, Ulster University, Coleraine Campus, North Ireland, UK; University Hospital, Kwame Nkrumah University of Science and Technology, Kumasi, Ghana; School of Medicine and Dentistry, Kwame Nkrumah University of Science and Technology, Kumasi, Ghana; School of Pharmacy and Pharmaceutical Sciences, Ulster University, Coleraine Campus, North Ireland, UK; School of Public Health, Kwame Nkrumah University of Science and Technology, Kumasi, Ghana; University Hospital, Kwame Nkrumah University of Science and Technology, Kumasi, Ghana; University Hospital, Kwame Nkrumah University of Science and Technology, Kumasi, Ghana; University Hospital, Kwame Nkrumah University of Science and Technology, Kumasi, Ghana; University Hospital, Kwame Nkrumah University of Science and Technology, Kumasi, Ghana; University Hospital, Kwame Nkrumah University of Science and Technology, Kumasi, Ghana; Department of Pharmacy Practice, Faculty of Pharmacy and Pharmaceutical Sciences, College of Health Sciences, Kwame Nkrumah University of Science and Technology, Kumasi, Ghana; School of Medicine and Dentistry, Kwame Nkrumah University of Science and Technology, Kumasi, Ghana; Department of Clinical Microbiology, Komfo Anokye Teaching Hospital, Kumasi, Ghana; Department of Pharmacy Practice, Faculty of Pharmacy and Pharmaceutical Sciences, College of Health Sciences, Kwame Nkrumah University of Science and Technology, Kumasi, Ghana

## Abstract

**Background:**

Antimicrobial stewardship (AMS) is imperative in addressing the menace of antimicrobial resistance (AMR) in health systems. Commonwealth Partnerships for Antimicrobial Stewardship uses a health partnership model to establish AMS in Commonwealth countries. The Hospital of Kwame Nkrumah University of Science and Technology in partnership with Ulster University, Northern Ireland, undertook an AMS project from November 2021 to May 2022. We report on implementation of the AMS, its impact on antibiotic use and infections management at the University Hospital; Kumasi, Ghana.

**Methods:**

The Global-Point Prevalence Survey (PPS) protocol was used to assess antibiotics use at the hospital at baseline, midpoint and end of the project. Feedback on each PPS was given to the hospital to inform practice, behavioural change and improve antibiotic use.

**Results:**

Antibiotic use reduced from 65% at baseline to 59.7% at the end of the project. The rate of healthcare-associated infections also reduced from 17.5% at baseline to 6.5%. Use of antibiotics from the WHO Access group was 40% at baseline but increased to 50% at the endpoint. Watch antibiotics reduced from 60% to 50% from baseline. Culture and susceptibility requests increased from baseline of 111 total requests to 330 requests in the intervention period to inform antimicrobial therapy

**Conclusion:**

The model AMS instituted improved antibiotic use and quality of antimicrobial therapy within the study period. Continuous staff education and training in AMS, and use of standard tools for assessment and application of local data to inform infections management will ensure sustenance and improvement in the gains made.

## Background

Low- and middle-income countries (LMICs) such as Ghana are known to have a high burden of antimicrobial resistance (AMR), which adversely impacts on healthcare delivery and patients’ outcomes.^1,2^ AMR is associated with significant morbidity and mortality in affected patients following infections from drug-resistant pathogens. The associated increase in healthcare costs in relation to the menace of AMR also places a great economic burden on the already constrained finances of households in LMICs.

In such LMICs, interventions aimed at improving antibiotic use need to be cost-effective to achieve a sustainable impact. A situational analysis of the AMR challenge in Ghana led to the identification of many gaps regarding the use of antibiotics in the health system and measures to combat this challenge.^3^ Subsequently, the development of Ghana’s first policy and National Action Plan guided by the Global Action Plan of WHO and partners, outlined strategies and interventions required to fight AMR in Ghana.^4,5^ The action plan was based on the one health concept, which takes a holistic approach to fight AMR. It advocates for increased surveillance on AMR and antimicrobial use (AMU), infection prevention and optimized AMU across the human, animal and food production, and environmental health sectors.^6^

Antimicrobial stewardship (AMS) is known to be a cost-effective means of reducing the progression of AMR especially in resource-limited settings with a high AMR burden.^7–10^ AMS programmes enable health facilities to optimize antibiotic use and improve health outcomes from infectious disease management. Impactful AMS programmes should possess important core elements and structure to be successful and ensure a long-term and sustainable impact. The WHO has developed a practical toolkit based on which institutions in LMICs can model their programmes.^11^ Additionally, the Commonwealth Partnerships for AMS (CwPAMS), managed by the Commonwealth Pharmacists Association and Tropical Health and Education Trust, uses a health partnership model shown to be effective in establishing AMS programmes in LMICs in Commonwealth countries.^12^ This model leverages expertise from the National Health Service facilities in the UK to support the implementation of AMS in LMIC Health facilities. CwPAMS, based on this model, has also developed a toolkit to support institutions to set up sustainable cost-effective programmes to improve antibiotic use.^13^

Core among AMS activities are prevalence surveillance of antibiotic use.^14,15^ Point prevalence surveys (PPS) on antibiotic use is an established standard means of measuring antibiotic use among inpatients. Their relatively low cost of delivery means they are easy to conduct in LMIC health facilities.^16^ PPSs support AMS programmes to assess their impact on antibiotic use through multiple evaluations as well as identifying targets for improvement in antibiotic use.^17,18^

A previous assessment of antibiotic use through a PPS in hospitals in the Ashanti region including the University Hospital found antibiotic use to be relatively high among inpatients with potential implications for quality of patient care in infectious disease management.^18^ On the basis of the health partnership model established and gaps identified in the earlier study, the University Hospital of Kwame Nkrumah University of Science and Technology (KNUST) in collaboration with the School of Pharmacy, Ulster University, Coleraine Campus in Northern Ireland, and other researchers at KNUST implemented and monitored an AMS programme over a period of 7 months. The purpose of this paper is to report on the outcome of the AMS model intervention implemented and its impact on antibiotic use.

## Methods

### Study setting

The University Hospital is a 135-bed district-level hospital located on the campus of the KNUST, Kumasi. It has a catchment population of more than 300 000. It provides general and specialist services to students of the university, staff and their dependents as well as private patients within the Oforikrom Municipality and greater Kumasi.

The hospital as part of this project set up a multidisciplinary committee responsible for undertaking activities in AMS made up of three pharmacists, a nurse (who also served as the hospital’s IPC focal person), three medical doctors (one is an infectious disease specialist), a hospital administrator and two laboratory scientists. The committee met at least monthly to discuss AMS activities conducted in the hospital. The AMS team that met at least twice a month consisted of two pharmacists, one nurse, two medical doctors and one laboratory scientist.

The health partnership was set up between KNUST and Ulster University to implement AMS in the University Hospital. Ulster University provided technical support and expertise in AMS implementation based on experience from the National Health Service in the UK. Lead partners from both institutions met regularly through online meetings before, during and after the implementation of the project that facilitated knowledge exchange and success. There was no AMS in the hospital before this.

### Study design

The AMS intervention run from 1st November 2021 to 31st May, 2022 and targeted staff from all health cadres in the hospital through hybrid training and education seminars to enable staff identify ways in which they could include AMS in their routine activities while creating awareness on AMR, AMS and IPC.

Audits of antibiotic use (PPS) were done and feedback was given to prescribers and pharmacists in the hospital through face-to-face meetings and by sharing on their various WhatsApp platforms. A report on AMS activities including audits were also shared with the hospital management as feedback.

The AMS team conducted and provided feedback to the hospital staff (especially prescribers) on antibiotic use through PPSs after the beginning, midpoint (fourth month after project start) and towards the end of the project (sixth month after project) to determine the impact of the interventions.

#### Prevalence and pattern of antibiotic use

The PPS was a cross-sectional survey that took place on the 16^th^ November 2021, 22^nd^ March 2022 and 5^th^ May 2022 at the University Hospital. The Global-PPS (G-PPS) (https://www.global-pps.com/ accessed on 1 November 2021) protocol was used to measure antibiotic use at the hospital, with all patients who were already admitted early in the morning (before 8:00 a.m.) on the survey day being surveyed using the data collection forms. The Global-PPS protocol was used to conduct the PPS due to its ease of use and quick feedback that could be promptly used to assess stewardship activities as well as provide information to prescribers especially on prescribing patterns. Data were collected anonymously from both paper-based medical records as well as the Electronic Medical Records system on the wards. No patients or prescriber’s identifiers were collected during survey points. There was no direct interaction with patients. Unless a patient did not meet the inclusion criteria, every patient on the ward at the time of the survey was included in the PPS. All acute care inpatient wards, admitted patients in the ward as at 8:00 a.m. of survey day and antibiotics administered via oral, parenteral, rectal or inhalational routes were included in the study.

#### Healthcare-associated infection surveillance

The G-PPS has an optional healthcare-associated infection (HAI) module that enables a hospital to survey HAIs in greater detail.^19,20^ This module collects additional variables in addition to the main protocol variables to survey HAIs. The online data entry platform of the G-PPS analyses all these data to ascertain the HAI rate for each participating hospital.

### Data management and analyses

Data collected after each survey were sorted and structured to avoid data entry mix-ups. All data collected were stored on a password-protected and encrypted hospital desktop computer with access restricted to only the AMS team conducting this study. The study's data was put into the Global-PPS programme, an internet-based application for anonymized data entry, validation and reporting. Data were analysed and presented using median, frequencies, percentages and tables.

### Ethical considerations

#### Institutional review board statement

Approval for this project was obtained from the management of the University Hospital, KNUST. Ethical clearance was sought and obtained from the Committee on Human Research, Publications, and Ethics of KNUST. (CHRPE/AP/470/22 of 15 August 2022). Regarding data confidentiality, the electronic databases are kept in a password-protected computer belonging to the principal investigator. No patient identifiers were used in this study.

#### Informed consent statement

As this was a record review study with no patient identifiers, the issue of informed patient consent did not apply.

#### Data availability statement

Requests to access these data should be sent to the corresponding author. Additionally, data collected in this study were recorded using the G-PPS online tool and anonymously accessible on registration.

### AMS intervention

The intervention comprised of a bundled stewardship programme embedded with infection prevention and control involving an education, audit and feedback (information) strategy for improving antibiotic use at the University Hospital, based on the WHO AMS and Commonwealth Partnerships for Antimicrobial Stewardship (CwPAMs) toolkits. These are practical toolkits on which AMS programme can be modelled for impact and sustainability.^11,13^

The bundled stewardship programme involved the following;

- Education (capacity building)
  a. AMS/AMR/optimal antibiotic use training seminars
  b. IPC training seminar
- Audit and feedback (information)
  c. PPSs to assess antibiotic use
- Advocacy on rational prescribing
  d. Culture and drug susceptibility testing
  e. Therapy review by pharmacists and AMS team

#### Key stages of intervention

- In conjunction with the management of the University Hospital, a multidisciplinary AMS committee was formed as a stand-alone committee.
- Members of this committee constituted the AMS team that implemented various components of AMS and also conducted surveillance (PPS) on antibiotic use at the hospital.
- The team led by a Family Medicine Consultant and infectious disease specialist and a clinical pharmacist/researcher subsequently carried out daily reviews of antibiotic use on the ward by pharmacists, monthly AMS team meetings and quarterly stewardship activities to achieve the objectives of the project. AMS activities carried out included the following:
  - Monitoring to ensure indications for antibiotic therapy are appropriately documented.
  - Educating clinicians to take samples for culture and susceptibility analyses before empiric antibiotic therapy is initiated.
  - Conduct training seminars to provide counselling and recommendations to other clinicians to improve prescribing behaviours through seminars and ward rounds.
  - In consultation with infectious disease specialist and other consultants in the hospital, selecting antibiotics to place on restricted access (requiring preauthorization before use) to reduce their inappropriate use.

## Results

### Demographic characteristics of patients

A total of 152 patient records were included in the three PPSs conducted. Most of the patients involved in the study were female (63.2%). The median age was 26.5 years (Table 1).

**Table 1.** Demographic characteristics of patients prescribed antibiotics at the University Hospital, KNUST.

Variables	Total *n* = 152 (%)	PPS 1 *n* = 46 (%)	PPS 2 *n* = 48 (%)	PPS 3 *n* = 58 (%)
Sex				
Male	56 (36.8)	20 (43.5)	16 (33.3)	20 (34.5)
Female	96 (63.2)	26 (56.5)	32 (66.7)	38 (65.5)
Age group (years)				
<2	10 (6.6)	2 (4.3)	4 (8.3)	4 (6.9)
2+	142 (93.4)	44 (95.7)	44 (91.7)	54 (93.1)
Median age (Q1, Q3)	26.5 (19, 38)	43.5 (24, 64)	21.0 (11, 27)	26.0 (19, 34)

### Prevalence of antibiotic use, prescription patterns and invasive device use

Antibiotic use at baseline was 65%, and reduced to 59.7% at the end of the project. Intravenous (IV) therapy reduced from 70.8% to 64.7%. The HAIs rate also reduced from 17.5% at baseline to 6.5% (Table 2).

**Table 2.** Prevalence, pattern of antibiotics and invasive device use at baseline and project endpoint in the University Hospital, KNUST.

Parameter	Baseline (%)	Midpoint (%)	Endpoint (%)
Patient prescribed at least one antibiotic	26 (65)	29 (56.9)	37 (59.7)
IV therapy	17 (70.8)	16 (59.3)	22 (64.7)
Patient on multiple antibiotics	14 (58.3)	10 (37)	13 (38.2)
HAIs rate	17.5	5.9	6.5
Peripheral vascular catheter	37 (92.5)	46 (90.2)	49 (79)
Indwelling urinary catheter	6 (15)	5 (9.8)	6 (9.7)
Tubes/drains	2 (5)	1 (2)	2 (3.2)

### Antibiotic classes used among inpatients

At baseline and endpoint, third-generation cephalosporins were the most used antibiotics. At midpoint, penicillin with beta-lactamase inhibitors were the most used at 21.4% (Table 3).

**Table 3.** Distribution of antibiotic classes used among inpatients at baseline and project endpoint in the University Hospital, KNUST.

Antibiotic classes prescribed	Baseline (%)	Midpoint (%)	Endpoint (%)
Penicillin with extended spectrum (Piperacillin/tazobactam)	4.8	0	3.8
Penicillin with beta-lactamase inhibitors (Amoxicillin/clavulanic acid)	11.9	21.4	17.3
Second-generation cephalosporins (cefuroxime)	11.9	9.5	13.5
Third-generation cephalosporins (ceftriaxone, cefixime)	21.4	14.3	21.2
Carbapenems (meropenem)	2.4	0	1.9
Macrolides (azithromycin)	11.9	7.1	3.8
Lincosamides (clinidamycin)	11.9	9.5	9.6
Fluoroquinolones (ciprofloxacin, levofloxacin)	11.9	7.1	9.6
Imidazole derivatives (metronidazole, tinidazole)	11.9	14.3	9.6
Aminoglycoside (gentamicin, amikacin)	0	7.1	7.7
Sulfonamide/trimethoprim	0	4.8	0
Tetracyclines (doxycycline)	0	2.4	0

### Indications for antibiotic use

Table 4 shows the indications for antibiotic use in University Hospital, KNUST during the intervention. At baseline, lower respiratory tract infections were the highest indication for antibiotic use at 40.9%. At endpoint, 14.3% of indications were lower respiratory infections and upper urinary tract infections.

**Table 4.** Indications for antibiotic use among patients prescribed antibiotics at baseline and project endpoint in the University Hospital, KNUST.

Indication	Baseline *n* (%)	Midpoint *n* (%)	Endpoint *N* (%)
Lower respiratory tract infections	9 (40.9)	4 (19)	3 (14.3)
Lower urinary tract infection	4 (18.2)	2 (9.5)	4 (19)
Skin and soft tissue infection	4 (18.2)	3 (14.3)	2 (9.5)
Sepsis	2 (9.1)	1 (4.8)	4 (19)
Central nervous system infections	1 (4.5)	2 (9.5)	1 (4.8)
Gastrointestinal infections	1 (4.5)	3 (14.3)	1 (4.8)
Upper urinary tract infections	1 (4.5)	2 (9.5)	3 (14.3)
Obstetric/gynaecological infections	0	2 (9.5)	1 (4.8)
Ear, nose and throat infections	0	1 (4.8)	1 (4.8)
Bone/joint infections	0	1 (4.8)	0
Bronchitis	0	0	1 (4.8)

### Antibiotic use distribution according to WHO AWaRe classification

**Figure 1.**
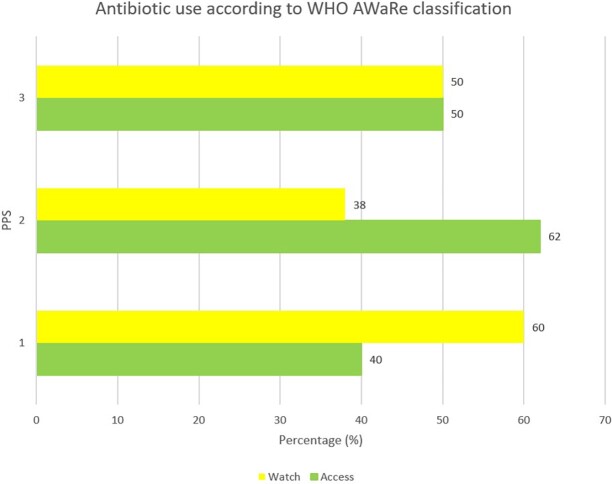
Antibiotic use among inpatients at the University Hospital, KNUST according to the WHO AwaRe classification at the project endpoint.

Antibiotic use according to WHO’s AWaRe classification in the University Hospital, KNUST is presented in Figure 1. None of the antibiotics prescribed belonged to the Reserve category. At baseline (PPS 1) Access antibiotic use was 40% but increased to 62% at midpoint and finally levelled at 50% at the project endpoint (PPS 3).

### Culture and drug susceptibility testing requests

**Figure 2.**
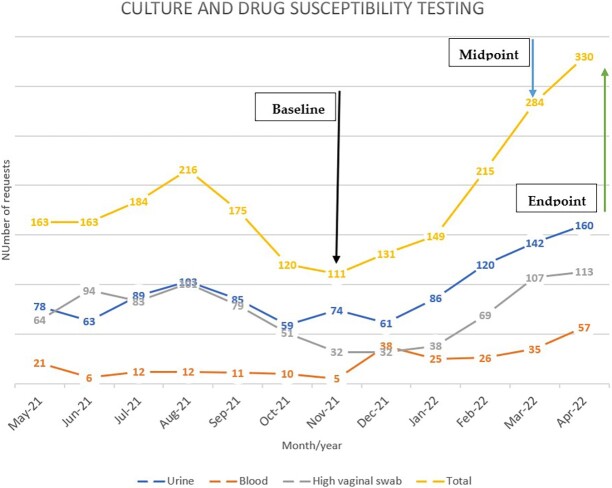
Culture and drug susceptibility request pattern at University Hospital, KNUST.

Culture and drug susceptibility testing (CDST) requests increased from the beginning of the intervention in November 2021 with 111 total requests to 330 requests as at April 2022. The pattern of requests is provided in Figure 2. Urine cultures accounted for the largest proportion of CDST requests.

## Discussion

The multiple PPSs demonstrated the impact of the AMS programme instituted at the University Hospital. To the best of our knowledge, this is the first report from Ghana on the impact of AMS on antibiotic use among inpatients at a hospital. At baseline, about two-thirds of patients were on antibiotics. This is more than twice the WHO optimum for rational antibiotic use in hospitals (20%–27%).^21^ Over the period of the intervention, the prevalence of antibiotic use initially reduced by 8% but went up by 2.8% at the end, which was still lower than at baseline. This is comparable to a national AMS project in China over a year (2011–2012), which reported a reduction in antibiotic use from 68% to 58% among inpatients.^22^ Prevalence of antibiotic use in this project was, however, higher at the endpoint than in a project from Nigeria where antibiotic use reduced from 82.5% at baseline, 68.3% at midpoint and 51.1% at endpoint.^23^ The Nigerian project, however, ran between 2015 and 2018 compared to the current project that implemented AMS over a 7-month period. Continued AMS activities may further reduce the prevalence of antibiotic use in the hospital. The prevalence of antibiotic use in this study was also higher than that from other Ghanaian hospitals (55%) where PPS was done and subsequently efforts have been made to implement AMS.^24^ Similar studies in Ghana evaluating antibiotic use in hospitals were conducted to assess opportunities for AMS, unlike the current study that assessed the impact of post-AMS deployment on antibiotic use.^18,25,26^ A high baseline prevalence of antibiotic use could be attributed to the COVID-19 pandemic that resulted in increased antibiotic use worldwide among COVID-19 patients.^27^ The pandemic may have affected prescriber behaviours relating to antibiotic use, although this may not always have been in a negative way due to differing health system characteristics.^28^ The initial reduction in antibiotic use at the hospital (midpoint) is likely to be a result of the feedback provided to prescribers after each PPS as a means of creating awareness to improve prescribing. Additionally, the novelty of the AMS programme as well as the engagement surrounding it may have driven the initial drop in antibiotic use. The marginal increase between the second and third PPS could be attributed to a possible increase in infectious diseases during that time leading to increased antibiotic use. To ensure sustainability of the AMS activities at the University Hospital, further research is needed to understand the possible mechanisms behind the change in antibiotic use. This may include a qualitative study to assess perceptions and behavioural factors among prescribers relating to antibiotic use. Although this is higher than WHO’s optimum, it is a demonstration of the potential of AMS programmes to improve antibiotic use in hospitals. Antibiotic use overall seemed to have improved during this period of the surveys. The AMS programme at KNUST needs to be sustained to further improve antibiotic use in the long term. Continuous and regular audits of antibiotic use, training of staff and implementation of evidence-based guidelines can help to sustain the programme.

The use of WHO’s Access antibiotics increased at the midpoint of the project by 22 points and decreased by 12 points at the endpoint. Watch antibiotic use decreased by 10 points to 50% of all antibiotic prescriptions at the end compared to baseline. Although improved from baseline, there is room for improvement as WHO recommends Access antibiotics to be at least 60% and other categories below 40%.^29^ Watch antibiotics are at increased risk of selection and development of resistance compared to Access antibiotics. Reducing their use among inpatients can objectively improve antibiotic prescribing. This is a key target for improvement in antibiotic use at the University Hospital as part of the ongoing AMS. This could be addressed by the implementation of AMS policies and guidelines for antibiotic use as well as the development and use of facility-specific antibiotic formularies.

An encouraging finding during the period was the increased number of CDST requests between the project start date and the end point. Requests for CDST more than doubled. Increased reliance on microbiology data from CDSTs are essential to improve antibiotic use and reduce AMR. They are also invaluable to supporting the implementation of AMS. Culture positivity rate as well as turnaround times for requests were not assessed. Such assessments could help to identify quality improvement of microbiological services as well as assess the capacity of the microbiology lab to handle such increased requests.

IV therapy was high but reduced at the endpoint. IV therapy requires the use of invasive devices, a potential source of HAI among inpatients. To optimize their use in the hospital, an IV to oral switch by pharmacists could be included in the bundled programme as it contributes to reduced use of IV therapy and consequent reduction in HAIs.^30^ At baseline, about a fifth of infections were considered healthcare associated whereas at the endpoint, HAIs reduced by 11 points. Such infections are more difficult to treat requiring improved IPC to prevent and control their spread.^31^ Training provided during the intervention period on AMR and IPC may have contributed to the reduction in HAIs observed.

To sustain the impact of this programme, a long-term action plan on AMS was developed as a result of this partnership to ensure continuity. Continuous AMS activities such as those used in this report are planned to ensure this.

### Strengths and limitations

The study has the following strengths. This intervention was implemented based on WHO’s AMS toolkit, which is based on international best practice for implementing AMS. The study was conducted and reported in accordance with the Strengthening the Reporting of Observational Studies in Epidemiology (STROBE) guidelines statement.^32^ However, there are some limitations to this study; no assessment of patient outcomes was conducted and the period over which these assessments were done was too short to assess the effectiveness of the programme. Antimicrobial consumption assessment was undertaken by using mainly the Global-PPS protocol. Future assessments could include antimicrobial consumption by a defined daily dose in addition to assessing patient outcomes and the AMR situation to allow further improvements to the AMS at the University Hospital, KNUST.^33–35^ Additionally, only three time points were evaluated at different months of the year that may be confounded by seasonal variations in antibiotic use. This may, however, not be significant as a recent assessment of antibiotic prescribing in the outpatient department of the hospital showed no seasonal variations in antibiotic use.^36^ Furthermore, the baseline demographic characteristics of patients as well as differing sites of infection, which may be a result of the intervention, precluded further analysis from being carried out.

### Conclusions

The AMS programme instituted at the University Hospital contributed to significant reduction in antibiotic use among inpatients over the intervention period. However, there was a marginal increase of about 3% in the prevalence of antibiotic use between the midpoint and end of the study evaluation. There was also an improvement in reduction of the rate of HAI as well as the consumption of antibiotics belonging to the Watch group in the WHO’s AWaRe classification at the University Hospital compared to baseline. Continuous staff education and training in AMS, regular use of quality improvement tools such as those from WHO for impact assessment and the application of local data to inform efficient therapy of infections will ensure sustenance in the gains made. This will thus minimize the risk of the emergence and spread of AMR with optimal health outcomes from infectious disease therapy.
